# How Important Are Structural Variants for Speciation?

**DOI:** 10.3390/genes12071084

**Published:** 2021-07-17

**Authors:** Linyi Zhang, Radka Reifová, Zuzana Halenková, Zachariah Gompert

**Affiliations:** 1 Department of Biology, Utah State University, Logan, UT 84322, USA; zach.gompert@usu.edu; 2 Department of Zoology, Faculty of Science, Charles University, 12800 Prague, Czech Republic; radka.reifova@natur.cuni.cz (R.R.); zuzka.halenkova@gmail.com (Z.H.)

**Keywords:** reproductive isolation, hybridization, suppressed recombination

## Abstract

Understanding the genetic basis of reproductive isolation is a central issue in the study of speciation. Structural variants (SVs); that is, structural changes in DNA, including inversions, translocations, insertions, deletions, and duplications, are common in a broad range of organisms and have been hypothesized to play a central role in speciation. Recent advances in molecular and statistical methods have identified structural variants, especially inversions, underlying ecologically important traits; thus, suggesting these mutations contribute to adaptation. However, the contribution of structural variants to reproductive isolation between species—and the underlying mechanism by which structural variants most often contribute to speciation—remain unclear. Here, we review (i) different mechanisms by which structural variants can generate or maintain reproductive isolation; (ii) patterns expected with these different mechanisms; and (iii) relevant empirical examples of each. We also summarize the available sequencing and bioinformatic methods to detect structural variants. Lastly, we suggest empirical approaches and new research directions to help obtain a more complete assessment of the role of structural variants in speciation.

## 1. Introduction

**Figure 1:**
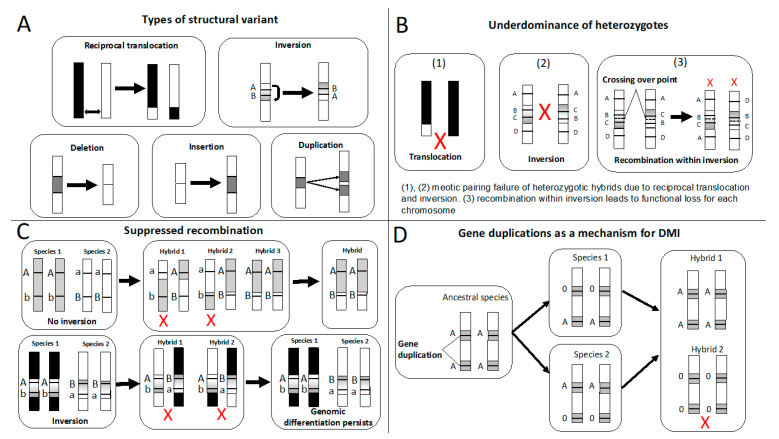
(**A**) Types of structural variants. (**B**) Underdominance of heterozygotes due to structural variants. Red Xs in (1) and (2) suggested meiotic pairing failure, while red Xs in (3) indicate reduced fitness of the individual. (**C**) Structural variants suppress recombination as a mechanism to prevent species from hybridizing. Allele a and b would cause hybrid inviability/sterility when present in the same genetic background. Without an inversion, recombination can break up the link between alleles at locus A and locus B and then selection can purge alleles a and b (see [14]). Red crosses indicate reduced fitness of the individual. (**D**) Gene duplications as a mechanism for DMI (Dobzhansky–Muller Incompatibilities). The 0 stands for loss of function at the locus. Red crosses indicate reduced fitness of the individual.

Identifying the genetic basis of reproductive isolation (RI) is important for understanding the speciation process, including how speciation begins and is completed [1,2,3,4]. Various genetic changes can contribute to RI and promote speciation. In some cases, mutations in a few genes are known to contribute substantially to RI, suggesting a relatively simple genetic basis for speciation. Such speciation genes have been linked to hybrid inviability in *Drosophila* (*Hmr*) [5], melanoma formation in *Xiphophorus* (*Xmrk-2*) [6], and changes in the mating system in *Solanum* spp. (*STYLE2.1*) (reviewed by [7,8,9]). However, speciation often involves many genes [10], and details of the genetic architecture of RI, including the types of mutations involved, could be critical for understanding the speciation process. The theory suggests that structural variants (see Figure 1A); that is, a type of mutation that cause a change in chromosomal location (translocation), orientation (inversion), or copy numbers (deletion, insertion and duplication) might be particularly important for speciation, especially in preventing species from merging upon secondary contact [11,12,13]. Here, we review this body of theory about the ways in which structural variants can contribute to RI, evaluate evidence in support of or against this theory, and discuss future avenues of productive research on the role of structural variants in speciation, with an emphasis on advances made possible by new molecular and statistical tools for detecting and analyzing structural mutations. Although SVs are highly variable in size, we focus on SVs greater than 50 bp in length [11].

The study of structural variants dates back to the early 1920s, when Alfred Sturtevant identified an inversion while comparing the chromosomes of *D. simulans* with its sister species *D. melanogaster* [15]. Since then, large structural variants have been identified using cytologic techniques in various species, including *Drosophila* [16], grasshoppers [17], and corn [18]. Due to the prevalence of structural variants in *Drosophila* and their easy identification with cytogenetic techniques, structural variants were among the first genetic markers used in population genetics [19]. Biologists in the 1930s–1960s hypothesized that these chromosome-level changes drove speciation based on the observation that many different structural variants were fixed between closely related species [17,20,21]. However, with the advent of other molecular markers (first allozymes, and then later microsatellites and single nucleotide polymorphisms [SNPs]) and an increased focus on the role of changes in gene expression versus protein sequences of individual genes, the emphasis on structural variants decreased some from the 1970 to the 1990s [1]. 

Recent advances in molecular and statistical methods have brought a renaissance in the study of structural variants, as these techniques can readily identify and genotype structural variants [22,23,24,25,26,27]. Recent evidence shows that large-scale structural variants, especially inversions, are associated with adaptive trait variation: including phenology in *Rhagoletis* fruit flies [3], mimetic wing patterns in *Heliconius* butterflies [28,29], cryptic coloration in *Timema* stick insects [30], the repeated evolution of distinct marine and freshwater ecotypes of three-spined sticklebacks [31], and ecotypes of *Helianthus* sunflowers [26]. However, in most cases, it is unclear whether the SVs associated with local adaptation represent balanced polymorphisms versus the early stages of speciation (or both). Here, we take advantage of these recent advances in the discovery and analysis of structural variants to review theory and evidence for structural variants causing RI and leading to speciation (Table 1), especially speciation with ongoing or episodic gene flow [3]. Then we discuss productive next steps and future directions for the study of structural variants, including highlighting methods for detecting and analyzing structural variants. 

## 2. Reproductive Isolation Caused by Structural Variants: Theory and Evidence

### 2.1. Underdominance of Heterokaryotypes (Hybrid-Sterility Models)

Structural variants such as inversions, translocations, and fusions were first proposed to promote reproductive isolation by producing sterile hybrids due to underdominance of the heterokaryotypes (i.e., reduced fitness of heterokaryotypes compared to homokaryotypes, Figure 1B, [62,63,64]). Specifically, mispairing of the rearranged chromosomes of heterozygotic individuals can prevent proper gametogenesis, leading to nonfunctional gametes. For example, Homolka et al. (2007) showed that being heterozygous for an autosomal translocation was responsible for male sterility in lab breeds of mice [65]. The incomplete synapses of the rearranged chromosome lead to meiotic silencing of unsynapsed chromatin. This process disturbs normally inactive single X chromosome in males, which causes male-limited sterility. As another example, Delneri et al. (2003) verified that a reciprocal translocation caused hybrid sterility among yeast by showing that fertility was recovered after engineering the genomic region to be collinear [66] (Table 1).

Whether sterility caused by underdominance of structural-variant heterozygotes is common in nature remains unclear [2,14,59,67]. Many studies have shown that the X chromosome has a large effect on male sterility [68,69,70,71]. However, the disproportionate effect of the X chromosome on male sterility is unlikely to be primarily caused by disruption of meiotic pairing of heterozygotes via structural variants, because males are hemizygous (X–Y chromosome) for the X chromosome [72]. It is noteworthy that the large-X effect can be caused by other mechanisms involving structural variants unrelated to the problems with meiotic pairing in heterozygotes, such as faster X evolution [73,74,75]. Another theoretical challenge for the underdominance-heterozygote model is that such variants are unlikely to become fixed in natural populations when their initial frequency is low because of the lower fitness of heterozygotes of structural variants. Specifically, the degree of under-dominance of a chromosomal rearrangement is inversely proportional to its probability of fixation, even though chromosomal rearrangement could be fixed by drift in a small population [76].

Interestingly, one form of translocation-centric fusions, which occur when two acrocentric chromosomes fuse to form a single metacentric chromosome, usually do not cause reduced fertility in heterozygous individuals within species [2]. However, the presence of different centric fusions in two species could cause sterility in interspecific hybrids if different species accumulate different centric fusions [33,77,78]. For instance, one population could experience a fusion of chromosomes 1 and 2, while another population experiences a fusion of chromosomes 1 and 3. Because both fusions involve chromosome 1 in this example, hybrids between populations will form quadrivalents or more complex multivalents during meiosis, causing sterility and promoting speciation [2]. One clear example of centric fusion contributing to speciation is in European house mouse *Mus musculus domesticus*. Different geographically localized races have different numbers of chromosomes, which is caused by the accumulation of different centric fusions [33,35,36,79] (Table 1). In the hybrid zone of these races, hybrids that are heterozygous for multiple centric fusions suffer a substantial loss of fertility. 

### 2.2. Suppressed-Recombination Models 

In light of the theoretical difficulties of the underdominance model described above, it was suggested that structural variants might be more likely to facilitate speciation by suppressing recombination rather than by directly reducing fitness of hybrids. For example, structural variants, especially inversions, can limit recombination among sets of alleles related to local adaptation and reproductive isolation, which can be particularly important for speciation with gene flow [14,67,80] (Figure 1C). Recombination is suppressed within inverted regions because of the mechanical problems during meiotic pairing [81], or because the recombined chromosomes often contain deleterious deletions or duplications leading to gamete inviability [67,82]. The effect of recombination suppression by inversions is supported by many studies across different taxa (e.g., *Helianthus* sunflowers [83], *Drosophila* [34,84,85,86,87], *Rhagoletis* [3], and fire ants [88]). Nonetheless, recent work has shown that gene conversion within inverted regions can at least partly homogenize the inverted sequence between the species even in the absence of recombination [89]. 

**Figure 2:**
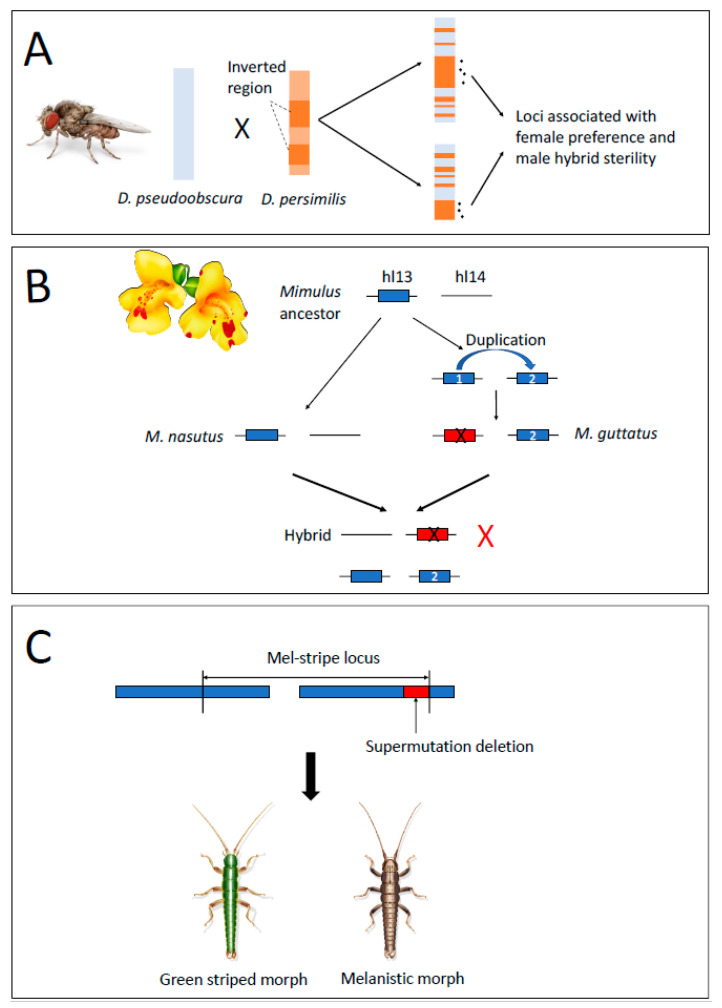
Example cases of structural variants involved in reproductive isolation. (**A**) Loci associated with female preference and male hybrid sterility located within an inversion in *Drosophila pseudoobscura* [14]. Blue color represents genomic region from *D. pseudoobscura*, and orange color represents genomic region from *D. persimilis*. (**B**) Loss of function in duplicated genes lead to hybrid lethality between *Mimulus guttatus* and *M. nasutus* [57]. Black crosses indicate the loss of the gene function, while the red cross represents the reduced fitness of the hybrid individual. (**C**) Deletion and inversion act as large mutation leads to color morph variation among *Timema* species both increasing (melanic morph) and decreasing (green versus striped morphs) RI between hosts [30]. Drawings of *Drosophila*, *Mimulus* and *Timema* credit to Rosa Marin Ribas.

Multiple underlying mechanisms have been proposed to explain how suppressed recombination promotes speciation. One of the earliest suppressed-recombination models was proposed by Rieseberg (2001) [67]. Specifically, Rieseberg (2001) argued that chromosomal rearrangements that suppress recombination could act synergistically with barrier genes to protect larger regions of the genome from introgression. As the number of regions of reduced recombination increases (e.g., as more inversions become fixed between nascent species), genome-wide differentiation could build up. Moreover, if genetic variants contributing to local adaptation and those contributing to other forms of reproductive isolation (e.g., assortative mating) are linked within an inverted genomic region, the progress of speciation could proceed more readily even with ongoing gene flow. Similar to the model proposed by Rieseberg (2001) [67], Noor et al. (2001) [14] suggested that inversions could promote speciation by reducing recombination across multiple linked loci each contributing to RI. This was suggested by Noor et al. (2001) after discovering that genomic regions associated with hybrid sterility and female species preference were clustered within two inverted regions of the genome that were fixed between two sister *Drosophila* species (*D. pseudoobscura* and *D. persimilis*, Figure 2A). Noor et al. posited that if two incompatible alleles that reduced hybrid fitness in one genetic background, or the other, are located within inversions, inversions would prevent the generation of viable hybrids via recombining genetic regions without incompatible alleles, thus maintaining species boundaries. Then, if the inversion(s) prevent species from fusing, there would be additional opportunities for linkage disequilibrium to build up between loci conferring hybrid inviability or sterility and those responsible for assortative mating, thereby completing the speciation process via reinforcement. 

A key aspect of the models proposed by Rieseberg 2001 and Noor et al., 2001 [14,67] is that SVs promote speciation by reducing introgression across a large block of the genome upon secondary contact, thus providing more time for loci conferring reproductive isolation to evolve and fix. However, they did not address: (1) whether genomic regions of suppressed recombination facilitate the fixation of loci conferring reproductive isolation; (2) whether suppressing recombination would favor the fixation of these structural variants between different species, especially in the face of gene flow. The above two questions were addressed via theoretical models by Navarro and Barton 2003 and Kirkpatrick and Barton 2006 [80,90], respectively. Navarro and Barton 2003 showed that genomic regions with reduced recombination rates promote the rapid fixation of independent mutations within each species that lead to DMIs between species, assuming chromosomal rearrangements had reached high frequency within species due to tight linkage to adaptive alleles. Later, Kirkpatrick and Barton 2006 showed that the condition of fixation of an inversion is quite general if it carries a multi-locus set of locally adaptive alleles. By suppressing recombination across the set of locally adaptive alleles, the inverted genomic region could have a fitness advantage relative to variants lacking the inversion and thus exhibiting higher rates of recombination. It is noteworthy that the selection advantage of an inversion depends on initial levels of recombination [91]. 

Recently, studies of SVs have found evidence consistent with the predictions from the hypothesis that SVs promote speciation by suppressing recombination. First, greater genetic differentiation between species has been found in inverted genomic regions than in non-inverted regions in fish [53,92], insects [3,25,85,93] and plants [55]. Second, studies have shown that inversions are more common in sympatric species than in allopatric species in both *Drosophila* [14] and passerine birds [94]. This is consistent with the prediction that hybridization results in selection for the spread of inversions in populations because specific combinations of alleles on the same chromosome are favored and inversions can minimize recombination of incompatible alleles from different species. Third, studies in European corn borer moths and *Drosophila* suggest that multiple adaptive or barrier loci occur within inverted regions [95,96], consistent with predictions from Kirkpatrick and Barton (2006). Last, genomic analyses suggest that adaptive alleles likely predated the origin and spread of inversions in the plant *Boechera stricta* [97,98], consistent with the prediction that newly emerged inversions can quickly spread and fix within species if they capture preexisting adaptive loci [90]. One caveat in *Boechera stricta* system is that since the inbreeding rate is high in the selfing system, the selective advantage of recombination suppression is low, and thus not sufficient to explain the fixation of the inversion [91]. 

One possible case of an inversion promoting reproductive isolation is found in *Anopheles gambiae* (mosquito) ecotypes [52] (Table 1). A rare 2Rj inversion is fixed in one ecotype of *A. gambiae* that specializes on rock pools as breeding sites. The researchers simulated the establishment and spread of this rare inversion given the realistic parameter ranges in *A. gambiae*, which is consistent with the speciation model proposed by Kirkpatrick and Barton 2006. They also found that the frequency of 2Rj inversion is highest among mosquitoes collected from rock pools, consistent with an adaptive role of the inversion to this specialized habitat. Lastly, they observed almost no heterozygotes for the 2Rj inversion despite no evidence of postzygotic isolation, suggesting (near) complete assortative mating between this ecotype and other *Anopheles* mosquitoes. 

While most studies have examined one or a few predictions made by the suppressed-recombination model, compelling evidence that SVs promote speciation by reducing recombination requires comprehensive analyses, showing that (1) structural variant frequency differs between reproductively isolated groups; (2) multiple barrier loci are found within the genomic region of structural variants; and (3) that loci within the structural variants contribute to reproductive isolation. One of the best cases suggesting that an inversion not only facilitates local adaptation, but also reproductive isolation involves the yellow monkeyflower (*Mimulus*) system [43,48] (Table 1). Lowry and Willis 2010 identified a chromosomal inversion associated with divergence between inland annual and coastal perennial ecotypes of *Mimulus guttatus* in key ecological traits including flowering time [48]. They further showed that reproductive isolation due to the trait differences associated with the inversion involved multiple reproductive barriers including immigrant inviability, temporal isolation, and extrinsic postzygotic isolation. Another study by Fishman et al., 2013 involving two sympatric sister monkeyflower species, *M. cardinalis* and *M. lewisii*, mapped traits that contribute to reproductive isolation, such as flowering time and hybrid sterility, to three regions of suppressed recombination, one reciprocal translocation and two inversions [43]. However, unlike Lowry and Willis 2010, Fishman et al., 2013 did not link the inversion directly to quantitative estimates of the strength of reproductive isolation in nature. 

Despite some evidence that inversions contribute to speciation by suppressing recombination, counter examples and theory also provide evidence against this hypothesis. First, the efficacy of inversions in suppressing recombination depends on the size of the inverted region. For instance, increased genomic differentiation was detected within a large (10-megabase) inversion affecting color in *Timema cristinae* stick insect, but no evidence of increased genetic differentiation was documented for other, smaller inversions [25]. Likewise, an analysis of sympatric *Heliconius* species found no evidence of large inversions fixed between species, and concluded that species specific inversions were too small (less than 50 kb) to prevent recombination across genome, and thus unlikely to be central to maintaining species barriers in this system [99]. Second, theoretical models showed that even a very low level of recombination within inverted regions would result in the loss of accentuated differentiation in inverted regions [100]. Two recent studies have found that gene conversion within inverted genomic regions is pervasive in both intraspecific crosses and interspecific crosses [89,101]. This suggests that there are some molecular mechanisms that could decrease the efficacy of inversions in suppressing recombination. Lastly, while the Kirkpatrick and Barton model indicates inversions carrying adaptive alleles could fix within the population quickly, inversions carrying a mixture of adaptive and deleterious mutations could result in within species polymorphism, rather than between population divergence and speciation [29]. 

### 2.3. Gene Duplications as a Mechanism of Intrinsic Postzygotic Isolation

The hypothesis that gene duplications promote speciation, especially by causing intrinsic postzygotic isolation dates back to Haldane (1933) [102]. Gene duplications can cause intrinsic postzygotic isolation in two ways: 1) independent loss of the function on one duplicated gene across two species causing a subset of backcross or F2 hybrids to be sterile or inviable; and 2) sub-functionalization of the duplicated genes, where gene duplicates evolve different functions between different species, leading to the reduced function in hybrids [103,104] (Figure 1D). Since neither the functional loss nor sub-functionalization of a copied gene is inherently deleterious, this process could be common [103,104,105]. Moreover, gene duplication is a common genomic feature that exists broadly across taxa [106,107,108]. Thus, gene duplication could play a major role in speciation. Nonetheless, empirical evidence demonstrating loss of function or sub-functionalization of duplicated genes as the underlying cause of hybrid sterility or inviability remains rare mainly due to the lack of genetic information in non-model systems (Table 1, but see [5,109]), especially in naturally hybridizing populations (but see [57]). 

One example comes from *D. simulans* and *D. mauritiana*, where the gene *Odysseus,* which causes hybrid-male sterility, arose via duplication of the ancestral gene *unc-4m* [5]. *Odysseus* has undergone rapid evolutionary change in terms of its DNA sequence and expression levels, consistent with the hypothesis that gene duplication gave the newly duplicated genes flexibility to evolve new functions ultimately leading to postzygotic reproductive isolation. Another example of how gene duplication results in evolutionary flexibility is found in the plant *Arabidopsis thaliana*, where an essential duplicated gene occurs in different genomic locations in different *A. thaliana* accessions resulting in recessive embryo lethality in crosses [58]. 

The first example of gene duplication causing hybrid genetic incompatibility in species known to hybridize in nature is in sympatric monkeyflower species (Figure 2B). Zuellig and Sweigart (2018) identified the lack of a functional copy of the critical photosynthetic gene *pTAC14* as the underlying genetic mechanism of hybrid lethality. In *M. guttatus*, the ancestral copy of the gene is no longer expressed, while in *M. nasutus*, the duplicated copy is missing. Hybrids die when they are homozygous for the nonfunctional *M. guttatus* copy and missing the duplicate from *M. nasutus*. More tests of the contribution of gene duplication to reproductive isolation in naturally hybridizing species are clearly needed in order to assess the importance of gene duplication as a genetic mechanism promoting speciation. 

### 2.4. Structural Variants Act as Mutations of Large Effect Causing RI

In addition to the mechanisms described above, structural variants can have direct and immediate phenotypic effects that could contribute to speciation. The phenotypic effects of structural variants might be particularly large if 1) insertions or deletions encompass multiple genes affecting a trait; or 2) the breakpoints of structural variants, such as inversions, disrupt a reading frame or alter expression at a developmental switch gene [30,110,111]. Many insertions come from transposons, which carry strong promotors that may alter the expression of nearby genes. For example, the insertion of a large transposable element was shown to change the expression of nearby genes that lead to the industrial melanism in peppered moths [112], where a retrotransposon insertion was found to reduce the expression of a gene affecting premating isolation in a genus of songbirds [60]. Another example where structural variants function as a large effect mutation can be seen in sticklebacks [113], where deletions in the regulatory *Pitx1* gene leads to reduction of pelvic spines, which may be adaptive in the absence of high piscine predator pressure or specific water chemistry. When such mutations affect traits contributing to reproductive isolation, the structural variants can contribute to speciation, but this is not necessarily the case in either the moth or the stickleback examples (but see an example on an insertion reduces expression of one gene affecting prezygotic isolation among songbirds, [60]).

Regardless of the effect on reproductive isolation per se, it is important to note that structural mutations can simultaneously affect traits by altering recombination and by altering gene expression or protein structure. This is especially true for inversions, as the mutational process, giving rise to inversions can also create deletions at the breakpoints [30]. This was likely the case for structural variants affecting cryptic color in *Timema* stick insects (Figure 2C). Here, several linked genes affecting color (green versus brown) reside within a small inversion in some species, but in others these color pattern loci reside within a deletion polymorphism at the breakpoint of a second, larger (10 megabase pair) inversion. Thus, the initial inversion likely generated suppressed recombination between color morphs, whereas the deletion associated with the second larger inversion further enhanced the phenotypic differences between morphs. However, once again this example does not provide evidence that large-effect structural variant mutations contribute directly to speciation, as the green versus brown color morphs represent a within species polymorphism, and do not appear to be directly related to speciation [114].

## 3. Critical Knowledge Gaps and Future Directions

**Figure 3:**
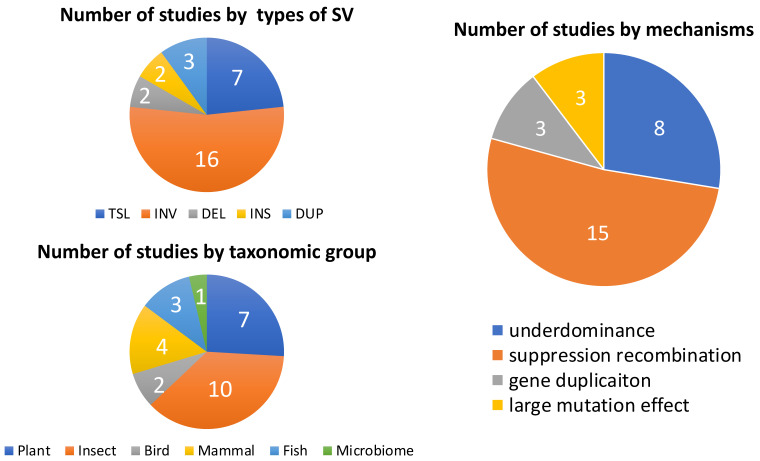
Summary of studies of structural variants involved in reproductive isolation. TSL, INV, DEL, INS and DUP stand for translocation, inversion, deletion, insertion and duplication, respectively.

We summarize a total of 27 studies identifying structural variants underlying reproductive barriers (Table 1 and Figure 3). Many case studies where structural variants causing adaptive phenotypic changes that could confer prezygotic isolation were not specifically connected to speciation (Table 1 and Figure 3). Since prezygotic reproductive isolation could be critical during the speciation process, especially for speciation-with-gene flow [115], future studies should quantify the relative strength of prezygotic reproductive barriers caused by structural variants compared to the total reproductive isolation between lineages. Furthermore, as many reproductive barriers, especially hybrid inviability and sterility could accumulate after speciation is complete [2], it is important to know whether the reproductive barrier caused by structural variants plays a role during the speciation process: from initiating speciation to maintaining species boundaries late in the speciation process.

Existing studies connecting structural variants to traits mostly focus on the effect of inversions in suppressing recombination among loci underlying one or a few traits conferring reproductive isolation (Figure 3). Such studies mainly concentrate on specific structural variants [3,14,116], a few reproductive barriers that are easy to identify (e.g., hybrid inviability or sterility, see [37,57], and a few model systems that are easy to conduct hybrid crossing in the lab (e.g., *Drosophila*; monkey flower *Mimulus*). While the effect of other types of structural variants on promoting reproductive isolation and speciation are less studied. Hybrid zones have long-served as natural laboratories for the study of speciation, and could be productively used to provide a powerful and more comprehensive framework for assessing the contribution of structural variants to speciation. Specifically, barriers to gene flow are tested in hybrid zones under natural conditions, and patterns of introgression across hybrid zones reflect, in part, the contribution of individual traits and gene regions to reproductive isolation [117,118,119,120]. Despite the power of hybrid zone analysis, few studies have investigated patterns of introgression for structural variants across hybrid zones (but see [13]). For example, structural variants could resist introgression across hybrid zones by preventing recombination among multiple barrier loci in inversions, by negatively affecting fitness by contributing to DMIs, or function as a large effect variant. Two patterns in hybrid zones that would suggest a disproportionate contribution of structural variants to reproductive isolation included reduced introgression (narrow clines) for (1) SNPs within structural variants (especially inversions) or (2) the structural variants themselves. 

Recent advances in molecular and statistical methods make widespread discovery and genotyping of structural variants more practical than ever. First, long-read sequencing technologies, such as nanopore sequencing and single molecule real-time sequencing, allow for easier detection of structural variants, especially complex structural variants, than earlier approaches, such as mate-pair sequencing with short reads (e.g., [121]). Specialized computer software for structural variant calling with these new read technologies is also advancing rapidly (Table 2). Alternative approaches that do not require long-read data exist for identifying and genotyping some types of structural variants. For example, principal component analysis of SNP genotype data can identify genomic regions with excessive population structure which may be caused by suppressed recombination within inversions [26,122], and short-read Illumina DNA sequence data have also proven useful to identify copy number variants [123,124]. Importantly, once structural variants are identified and structural variant genotypes have been estimated, these genetic markers can be analyzed in much the same way and with the same software and models as have been used for SNP data sets. The one caveat being that models incorporating error sources to accurately calculate genotype likelihoods have not yet been as well-developed for SVs as for SNP loci.

In conclusion, we now know that structural variants can, in principle, contribute to reproductive isolation by various mechanisms, but we do not know which of these are most important or about the relative importance of structural variants versus point mutations. Even though some studies have mapped phenotypic traits underlying reproductive barriers to structural variants, such as inversions, very few studies have distinguished whether structural variants affect RI by suppressing recombination of multiple adaptive alleles, or large effect of mutations, or other genetic mechanisms (but see [30,98]). Fine-scale genomic mapping and functional manipulation and validation of genes are necessary to tease apart the effects, such as changes in gene expression via breaking points vs. carrying functional genes within the structural variant [149]. 

Lastly, we need to move from a few cases in model organisms, to understand the contribution of SVs to speciation across taxa. Thus, more work is needed on the role of SVs in generating RI in cases of recent or ongoing speciation. Likewise, additional macroevolutionary studies are needed to evaluate the importance of structural variants along a deep evolutionary time (e.g., mammals [150], lizards [151], birds [94], and butterflies [152]). Only by combining the micro and macro-evolutionary patterns, can we achieve a holistic view of the importance of structural variants in promoting speciation. 

## Figures and Tables

**Table 1:** List of studies that identified structural variants involved in reproductive isolation.

Mechanism by Which SV Contributes to RI	SV Type	Study Organism	Affected RI	References
Underdominance	TSL	Sunflower hybrid species and their parental species (*H. annuus* and *H. petiolaris*)	Pollen sterility in hybrids.	Lai et al., 2005 [32]
Underdominance	TSL	House mouse *Mus musculus domesticus*	Hybrid subfertility and sterility	Hauffe and Searle 1993 [33]; Nachman and Searle 1995 [34]; Pialek et al., 2008 [35]; Garagna et al., 2014 [36]
Underdominance	TSL, INV	Fission yeast *Schizosaccharomyces pombe*	Intrinsic hybrid inviability	Jeffares et al., 2017 [37]
Underdominance	TSL, INV	Red brocket deer *Mazama americana*	Hybrid subfertility and sterility	Abril et al., 2010 [38], Cursino et al., 2014 [39]
Underdominance	DEL	Mountain pine beetle *Dendroctonus ponderosae*	Hybrid male sterility	Bracewell et al., 2017 [40], Dowle et al., 2017 [41]
Underdominance	INS	*Fruit flies Drosophila simulans* and *D. melanogaster*	Hybrid lethality	Ferree and Barbash 2009 [42]
Suppressed recombination and underdominance of heterokaryotypes	INV, TSL	Monkeyflowers *Mimulus cardinalis* and *M. lewisii*	Ecological isolation caused by adaptation to different elevation ranges and pollinators. Hybrid sterility.	Fishman et al., 2013 [43]
Suppressed recombination and underdominance of heterokaryons	INV	Mosquito *Anopheles funestus*	Assortative mating and postzygotic isolation resulting from underdominance of heterozygotes in particular habitats.	Ayala, Guerrero and Kirkpatrick 2013 [44]
Suppressed recombination and possibly underdominance of heterokaryons	TSL	Killifish *Lucania goodei* and *L. parva*	Hybrid inviability, behavioral isolation	Berdan, Fuller and Kozak 2021 [45]
Suppressed recombination	INV	Stick insect *Timema cristinae*	Habitat isolation associated with different cryptic color patterns (specialization to different host plants).	Nosil et al., 2018 [46], Lucek et al., 2019 [25]
Suppressed recombination	INV	Deer mouse *Peromyscus maniculatus*	Ecological isolation (local adaptation to different environments).	Hager et al., 2021 [47]
Suppressed recombination	INV	Three-spined stickleback (*Gasterosteus aculeatus*)	Ecological isolation caused by adaptation to freshwater and marine environment.	Jones et al., 2012 [31]
Suppressed recombination	INV	Yellow monkeyflower *Mimulus guttatus*	Ecological isolation including temporal isolation and habitat isolation.	Lowry and Willis 2010 [48], Oneal et al., 2014 [49], Twyrord and Friedman 2015 [50]
Suppressed recombination	INV	Apple maggot fly *Rhagoletis pomonella*	Ecological isolation including temporal isolation caused by adaptation to different host plants.	Feder et al., 2003 [51]
Suppressed recombination	INV	Fruit flies *Drosophila pseudoobscura* and *D. persimilis*	F1 hybrid male sterility, backcross hybrid inviability, F1 hybrid male courtship dysfunction, female species-specific preferences	Noor et al., 2001 [14]
Suppressed recombination	INV	Mosquito *Anopheles gambiae*	Ecological isolation caused by divergence in breeding sites.	Manoukis et al., 2008 [52]
Suppressed recombination	INV	The Northeast Arctic cod and Norwegian coastal cod populations of the Atlantic cod	Ecological isolation caused by different migration behaviors.	Kirubakaran et al., 2016 [53]
Suppressed recombination	INV	Long-tailed finch *Poephila acuticauda*	Unknown. Potentially prezygotic isolation caused by differences in bill color or sperm morphology between species.	Hooper et al., 2019 [54]
Suppressed recombination	INV	Prairie sunflower *Helianthus petiolaris*	Ecological isolation caused by adaptation to different environments	Huang et al., 2020 [55]
Suppressed recombination	INV	Fruit flies *Drosophila mojavensis* and *D. arizonae*	Unclear	Lohse et al., 2015 [56]
Suppressed recombination	INV	Sunflower species *Helianthus annuus*, *H. petiolaris* and *H. argophyllus*	Ecological isolation including temporal isolation caused by different flowering times	Todesco et al., 2020 [26]
Change of gene position caused by gene duplication and functional loss of ancestral copy in one species.	DUP	Monkeyflowers *Mimulus guttatus* and *M. nasutus*	F2 hybrid inviability	Zuellig and Sweigart 2018 [57]
Ancestral gene duplication has predisposed one of the paralogs for fast evolutionary rates.	DUP	Fruit flies *Drosophila mauritiana* and *D. simulans*	Hybrid male sterility	Ting et al 2004 [5]
Reciprocal gene loss after duplication	DUP	Wild strains of the plant *Arabidopsis thaliana*	Hybrid lethality in F2 cross	Bikard et al., 2009 [58]
Change of gene position	TSL	Fruit flies *Drosophila melanogaster* and *D. simulans*	F2 hybrid sterility	Masly et al., 2006 [59]
Mutations induced by SV	INS	Crow subspecies *Corvus corone corone* and *C. c. cornix*	Premating isolation	Weissensteiner et al., 2020 [60]
Source of mutations	DEL	House mouse *Mus musculus musculus* and *M. m. domesticus*	Assortative mating in the secondary contact zone (reinforcement)	North et al., 2020 [61]

**Table 2:** Methods to detect structural variants.

Sequencing Method	Sequencing Platform	Alignment Method (Software)	Variant Calling (Software)	SV Types	Author (Study)
Long-read sequencing	ONT, PacBio	BWA-MEM [125], Minimap2 [126], NGMLR [127]	Sniffles	DEL, DUP, INS, INV, TRA, nested SVs (INVDUP, INVDEL)	Sedlazeck et al., 2018 [127]
	ONT, PacBio	Minimap2 [126], NGMLR [126]	SVIM	DEL, DUP, INS, INV, TRA	Heller and Vingron, 2019 [128]
	ONT, PacBio	Minimap2 [126], LAST [129]	NanoVar	DEL, INV, DUP, INS, TRA	Tham et al., 2020 [130]
	ONT, PacBio	BWA-MEM [125], Minimap2 [126], NGMLR [127], LAST [129]	NanoSV	DEL, INS, DUP, INV, TRA	Stancu et al., 2017 [131]
	PacBio	BLASR [132]	PBHoney	INS, DEL, INV, TRA	English et al., 2014 [133]
	PacBio	BLASR [132]	SMRT-SV	INS, DEL, INV	Huddleston et al., 2017 [134]
	ONT, PacBio	Minimap2 [126]	cuteSV	DEL, INS, DUP, INV, TRA	Jiang et al., 2020 [135]
	PacBio	PBMM2 [136]	PBSV	INS, DEL, INV, DUP, TRA	
Short-read sequencing	Illumina (short read sequencing platforms in general)	BWA-MEM [125]	LUMPY	DEL, DUP, INV, TRA	Layer et al., 2014 [137]
	Illumina (short read sequencing platforms in general)	BWA-MEM [125]	DELLY	DEL, DUP, INV, TRA	Rausch et al., 2012 [138]
	Illumina (short read sequencing platforms in general)	BWA-MEM [125]	Pindel	DEL, INS, INV, DUP, TRA	Ye et al., 2009 [139]
	Illumina (short read sequencing platforms in general)—paired-end sequencing reads	MAQ [140], BWA [141], NovoAlign [142], Bfast [143]	BreakDancer	DEL, INS, INV, TRA	Chen et al., 2009 [144]
	Illumina	Stampy [145], BWA [141], SMALT [146], MAQ [140]	IMR/DENOM	INS, DEL	Gan et al., 2011 [147]
	Illumina	Stampy [145], BWA [141]	Platypus	INS, DEL	Rimmer et al., 2014 [148]

## Data Availability

No new data were created or analyzed in this study. Data sharing is not applicable to this article.
